# Design of Tranilast-Loaded Cation-Type Contact Lens for Sustainable Ocular Drug Delivery

**DOI:** 10.3390/pharmaceutics17060712

**Published:** 2025-05-28

**Authors:** Toru Matsunaga, Ryotaro Kuwamura, Shiori Hino, Fumihiko Ogata, Hiroko Otake, Naohito Kawasaki, Shinichiro Kobayakawa, Noriaki Nagai

**Affiliations:** 1 Design and Development, SEED Co., Ltd., 1030-7 Fukuro, Kounosu-shi 369-0131, Saitama, Japan; toru_matsunaga@seed.co.jp (T.M.); s_hino@seed.co.jp (S.H.); 2 Faculty of Pharmacy, Kindai University, 3-4-1 Kowakae, Higashi-Osaka 577-8502, Osaka, Japan; 2211710045p@kindai.ac.jp (R.K.); ogata@phar.kindai.ac.jp (F.O.); hotake@phar.kindai.ac.jp (H.O.); kawasaki@phar.kindai.ac.jp (N.K.); 3 Department of Ophthalmology, Nippon Medical School, Musashi-Kosugi Hospital, 1-396, Kosugi-cho, Nakahara-ku, Kawasaki 211-8533, Kanagawa, Japan; s-kobayakawa@nms.ac.jp

**Keywords:** contact lenses, tranilast, autoclave, cation, lacrimal fluid

## Abstract

**Objectives:** This study evaluated the design of a sustained-release contact lens (CL) device loading tranilast (TRA) and determined the usefulness of these CLs in Japanese albino rabbits. **Methods**: The sustainable CLs in this study were prepared by combining three CLs with different water contents and soaking methods under high-pressure and high-temperature using an autoclave method (AC-method). **Results**: Both the CLs prepared with the conventional soaking method (stir-method) and AC-methods were transparent in all three types of CLs. The loaded TRA contents in the CLs when using the AC-method were higher than those prepared using the stir-method for all three types of CLs. TRA contents were also higher when loaded into the cation-type lenses as compared to the other lenses. Moreover, the sustainable release of TRA from the TRA-loaded cation-type CL using the AC-method was significantly higher than those found for the other CLs. No corneal wounds were observed in any of the rabbits given the three types of TRA-loaded CLs for 7 days. Furthermore, the TRA-loaded CL sustainably released TRA into the lacrimal fluid in the rabbit. **Conclusions**: The TRA-loaded CL prepared using the AC-method overcame the limitations normally associated with the stir-method, such as the high burst release and low drug uptake.

## 1. Introduction

Tranilast (TRA), N-[3,4-dimethoxycinnamoyl] anthranilic acid, prevents the allergy-associated enhancement of vascular permeability and the release of chemical mediators from mast cells [1,2]. Therefore, eye drops containing TRA are widely used as the antiallergy agent that is instilled for the treatment of allergic conjunctivitis [2]. Moreover, eye drops are the conventional form used in treatment, making up approximately 90% of the marketed ophthalmic formulations [3]. However, eye drops are often characterized by low sustainability and ocular bioavailability (BA), as the drug retention on the eye is limited by the blinking reflex, along with issues involving the renewal rate of the lachrymal fluid, the functioning of this sophisticated organ, and the high level of protection associated with the various biological barriers. Therefore, determining the best regulated sustainable release and ocular drug delivery system (DDS) has always been a significant challenge for scientists [4]. Recently, there have been many ocular DDSs that have been reported to be able to effectively deliver drugs to the ocular surface and anterior segment of the eye, including punctal plugs [5], intraocular contact lens [6], implants [7], mucoadhesive polymers [8], drug-eluting contact lenses [9,10,11,12,13], absorption enhancers [14], microneedles [15,16,17], nanoparticles [18], in situ gelling system [19], and iontophoresis [20]. Among these different systems, contact lenses (CLs) can be viewed as a potential strategy for enhancing the sustained release and ocular BA.

The most widely used method is the soaking method, which has been used to load ocular drugs in CLs [21], with this technique successfully used for loading ocular drugs, such as timolol maleate [22], dexamethasone [23], fluoroquinolones [24], and pilocarpine [25]. However, there are issues associated with the soaking method for drug loading in CLs, such as high burst release and low drug uptake [21]. In addition, it has been reported that CLs with built-in cyclodextrin (Cyc) released acetazolamide and ethoxzolamide for 24 h [26]. Moreover, CLs loaded with Cyc-ciprofloxacin and Cyc-dexamethasone were associated with a high burst release [27]. In contrast, it has also been reported that the affinity between a drug and CLs may be related to the drug loading [28]. A previous study demonstrated that a higher pilocarpine content was loaded to CLs when there was a lower water content as compared to when it was loaded to CLs when there was higher water content, with the loaded drug levels in the CLs with the lower water content exhibiting a 2-fold increase compared to the CLs with the higher water content after a 15 h soaking with 1% pilocarpine [25]. The results from these studies indicated that the main driving force for the release from the CLs and the drug delivery into the CLs depended on the difference in the drug concentration between the soaking solution and the aqueous phase of CLs, and the composition of the CLs related to the release profile and the loading of the drug in the device [29,30]. Many studies have been conducted to better elucidate this issue associated with CLs. Technologies, such as solvent casting, molecular imprinting, and nanoparticle incorporation, have been investigated to improve the sustained drug release properties of CLs [31,32,33]. However, due to the complexity of these fabrication processes, there remain some limitations with regard to the realization of disease treatment for the conventional soaking method, such as safety, production process, drug inclusion amount, and sustainability. Further research is required to enhance their practical applicability [31,32,33]. Therefore, we attempted to develop a drug-loading method for CLs that avoids complex procedures by employing the autoclaving process commonly used for sterilization (high-pressure and high-temperature conditions: a pressure of 15 pounds per square inch and a chamber temperature of 121 °C) in this study. Using this method, both the drug-loading capacity and the sustained release properties of the CL were enhanced. In addition, our findings indicated that the TRA-loading CLs when using the soaking methods under high-pressure and high-temperature using an autoclave (AC-method) improved the issues that previously limited the conventional soaking method (stir-method). These findings suggest the potential applicability of the device as a once-daily sustained DDS.

## 2. Materials and Methods

### 2.1. Reagents

Three types of CLs, which consisted of the non-ion-type, anion-type, and cation-type, were used in this study. All of the CLs were obtained from the SEED Co., Ltd. (Saitama, Japan). TRA powder was provided by Kissei Pharmaceutical Co., Ltd. (Nagano, Japan). Schirmer tear test strips were obtained from AYUMI Pharmaceutical Co. (Tokyo, Japan). One percent fluorescein and 0.4% Benoxil were purchased from Alcon (Tokyo, Japan) and Santen Pharmaceutical Co., Ltd. (Osaka, Japan), respectively. All other chemicals used were of the highest purity commercially available.

### 2.2. Animals

Japanese albino rabbits weighing 2.7 kg were purchased from Shimizu Laboratory Supplies Co., Ltd. (Kyoto, Japan), and used for the investigation of the drug release on the ocular surface. All of the rabbits were housed under standard conditions (light 7:00–19:00/dark 19:00–7:00, 25 °C), and were allowed free access to water and CR-3 commercial diet (Clea Japan Inc., Tokyo, Japan). All animal experiments were performed in accordance with the guidelines of ARVO, Kindai University, Guide for the Care and Use of Laboratory Animals provided by the National Institutes of Health, and Animal Research: Reporting of In Vivo Experiments (ARRIVE) guidelines, and were approved by the pharmacy committee for animal research of Kindai University (1 April 2022, project identification code KAPS-2022-011). Rabbits were placed in a holding device during the application of the CL, corneal observation, and sample collection. Every effort was made to minimize suffering by releasing the rabbits from the holding device as soon as possible after the application of the CL, corneal observation, and sample collection. Observations on the health of the rabbits and behavior were made daily.

### 2.3. Measurement of TRA Content

TRA concentrations in the in vitro studies were determined by using an Alliance HPLC system (HPLC, Waters Corporation, Milford, MA, USA) equipped with a 4.5 × 150 mm InertSustain^®^ C18 column (GL Science Co., Inc., Tokyo, Japan). All the volumes for each of the samples were evaporated to dryness using a nitrogen stream, re-dissolved in 2 mL methanol, and then injected into the HPLC. The column was eluted at 35 °C, with 50 mM ammonium acetate and acetonitrile (75:25) used as the mobile phase. The flow rate was 1.0 mL/min, with TRA detected at 335 nm.

### 2.4. Loading of TRA in the CLs

The CLs were added to phosphate-buffered saline (suspensions) containing 0.15% TRA (3 mL), with the loading of TRA in the CLs performed using two different methods. In the first method, the CLs were stirred for 24 h (method I, stir-method). All other processes were performed under high-pressure and high-temperature conditions (a pressure of 15 pounds per square inch and a chamber temperature of 121 °C) for 30 min using an autoclave (method II, AC-method). After these treatments, the CLs were lightly rinsed with phosphate-buffered saline, and then used for the experiments. Detection of the TRA contents in the CLs was performed after incubating the TRA-loaded CLs in 2 mL phosphate-buffered saline at 37 °C. After incubation for a predetermined time, the CLs were then placed into 2 mL phosphate-buffered saline for a predetermined time, with re-incubations repeated at 37 °C for up to 168 h. Furthermore, after re-incubating the CLs in 10 mL phosphate-buffered saline for 4 h or more, this process was then repeated 10 more times. Each of the phosphate-buffered saline samples was measured by the previously described HPLC method, with the loaded contents of the TRA in the CLs determined by adding up all of the results of the sample measurements.

### 2.5. Characteristics of TRA Suspensions

TRA characteristics were determined in accordance with the methods of our previous study [31,32,33]. The particle size of the suspensions containing TRA was measured by a laser diffraction particle size analyzer, Shimadzu SALD-7100 (Shimadzu Corp., Kyoto, Japan). The refractive index was set at 1.60–0.10 i. SEM images of the TRA were obtained using a NeoScope^TM^ JCM-7000 (JEOL Ltd., Tokyo, Japan). The zeta potential was obtained through the use of a Micro-Electrophoresis Zeta Potential Analyzer model 502 (Nihon Rufuto Co., Ltd., Tokyo, Japan).

### 2.6. In Vitro TRA Release from CLs

Two methods were used to evaluate the drug release from the CLs. In the first method, the profile of the drug release from CLs was measured using 2 mL phosphate-buffered saline (pH 7.2, isotonic) at 37 °C. After incubation for a predetermined time, the CLs were then transferred into 2 mL phosphate-buffered saline for a predetermined time, with the re-incubation repeated at 37 °C for up to 60 h. The second method was used to investigate the profile of the drug release from the pre-lens and post-lens of the CLs by using a methacrylate chamber designed for trans-corneal penetration studies [34,35,36]. The CLs were placed on a methacrylate cell, followed by the addition of 3 mL phosphate-buffered saline into both chambers (pre- and post-chambers). The experiments were performed at 37 °C for 6 h, with 50 µL of sample solution withdrawn from both chambers at the indicated times (0, 0.5, 1, 2, 3, 4, 5, 6 h). For both methods, the same volume of buffer was replaced at the same time when the sample solution was withdrawn. All TRA levels in the samples were measured by the previously described HPLC method. The area under the drug concentration–time curve (AUC_0–6 h_) was determined according to the trapezoidal rule, up to the last TRA concentration measurement point (6 h).

### 2.7. Image Analysis of Corneal Toxicity in Rabbits

The CLs were repetitively applied to the rabbits for 8 h a day (10:00–18:00) for 7 days. After 7 days, the corneal surfaces of the rabbits were also observed for 2 h after the application of the CLs by using the slit lamp METORI-50V (SEED Co., Ltd., Saitama, Japan) [non-ion (n = 5), anion (n = 5), and cation (n = 5)]. In addition, the corneas of the rabbits were washed with a solution containing 1% fluorescein and 0.4% Benoxil after obtaining the digital image, with the corneal wounds monitored using a METORI-50V equipped with a blue filter.

### 2.8. In Vivo TRA Release from CLs into Lacrimal Fluid

After the CLs were applied to the rabbits, the lacrimal fluid was collected using Schirmer tear test strips at 30 min, 1 h, 2 h, 4 h, and 6 h after the original application [non-ion (n = 5), anion (n = 5), and cation (n = 5)]. Subsequently, the collected Schirmer tear test strips containing the samples were homogenized in methanol in order to extract the TRA, with the TRA levels, then measured by the HPLC method. For the in vivo study, TRA concentrations were determined using an LC-20AT system (HPLC, Shimadzu Corp., Kyoto, Japan) equipped with a 2.1 × 50 mm Inertsil^®^ ODS-3 column (GL Science Co., Inc., Tokyo, Japan) [31,32]. After adding 10 µL of samples to 100 µL methanol containing 3 µg/mL ethyl p-hydroxybenzoate (internal standard), the samples were then injected into a Shimadzu HPLC LC-20AT system (Shimadzu Corp., Kyoto, Japan). The column was eluted at 35 °C, with 50 mM ammonium acetate and acetonitrile (80:20) used as the mobile phase. When the flow rate was set at 0.25 mL/min, TRA was detected at 230 nm. The area under the drug concentration–time curve in the lacrimal fluid (AUC_LF_) was determined according to the trapezoidal rule using all of the values determined up to the last TRA concentration measurement point (6 h).

### 2.9. Statistical Analysis

Statistical analysis was performed using JMP ver. 5.1 (SAS Institute, Tokyo, Japan). Data are expressed as the mean ± standard error (S.E.). Statistical analysis was performed using Student’s *t*-test and ANOVA followed by Dunnett’s multiple comparison, with *p* < 0.05 considered to be significant.

## 3. Results

### 3.1. Design of the TRA-Loaded CLs and Evaluation of Their Characteristics

**Figure 1:**
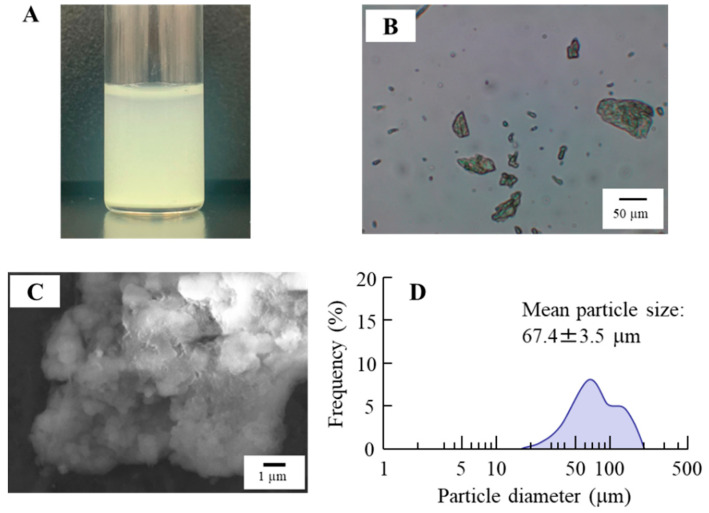
Image of digital (**A**), microscope (**B**), SEM (**C**), and particle size distribution (**D**) of the TRA suspensions. The particle size of the TRA suspensions was approximately 15–200 µm.

**Figure 2:**
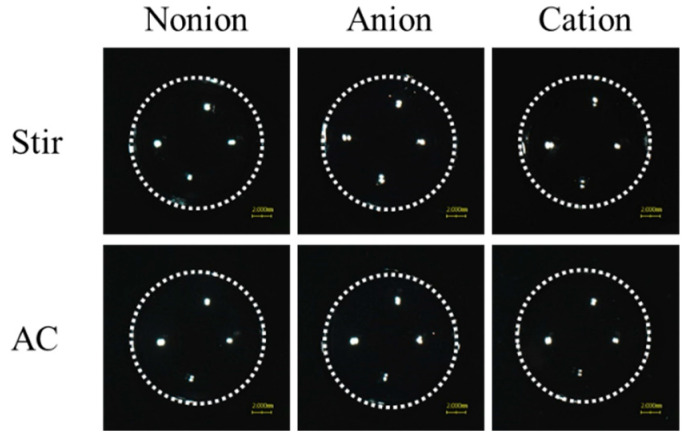
Digital image of the TRA-loaded CLs prepared using the stir- or AC-methods. There were three types of CLs used (non-ion-, anion-, and cation-type). The scale bar in the lower right corner of the photograph represents 2 mm. The TRA-loaded CLs prepared using the stir- and AC-methods were transparent in all three types of the CLs.

**Figure 3:**
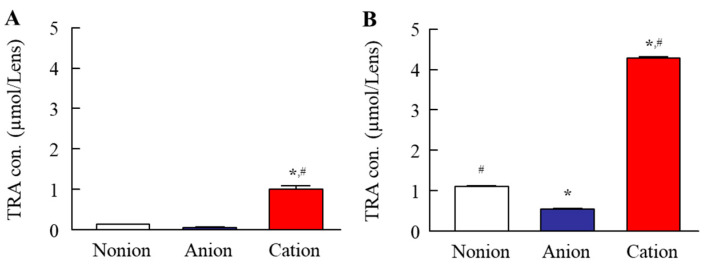
Total TRA contents for the TRA-loaded CLs prepared using the stir- (**A**) and AC-methods (**B**). There were three types of CLs used (non-ion-, anion-, and cation-type) (n = 3). * *p* < 0.05 vs. non-ion for each category. ^#^ *p* < 0.05 vs. anion for each category. The TRA contents were strongly loaded into the cation-type CL as compared to the other types of the CLs. Moreover, the loaded TRA contents in the CLs when using the AC-method were significantly higher than those found for the stir-method for all three types of CLs.

In this study, we attempted to load TRA into CLs using TRA suspensions. Figure 1 shows the characteristics of the TRA suspensions used. The color of this suspension was yellow, with the mean particle size of TRA in the suspensions was 67.4 ± 3.5 µm. The 0.15% TRA suspensions (3 mL in 5 mL test tube) precipitated within an hour, since the TRA concentration at a depth of 5 mm below the surface, measured one hour after preparation, was 0.02%. The zeta potential at that point was −67.6 mV. Figure 2 shows the images of the CLs after the loading of the TRA when using the stir- or AC-methods. All of the non-ion-, anion-, and cation-type CLs that were prepared using the stir- and AC-methods were transparent, with no deterioration in the CLs visually observed. We also measured the loaded TRA contents in the CLs using the stir- and AC-methods (Figure 3). Results showed that the AC-method loaded more TRA in the CLs as compared to that for the stir-method. In addition, the TRA levels in cation-type CLs were the highest compared with the non-ion- and anion-type CLs. The TRA levels in cation-type CLs were 3.8- and 7.9-fold higher than those in the non-ion- and anion-type CLs, respectively.

### 3.2. Drug Release from TRA-Loaded CLs to the Ocular Surface

**Figure 4:**
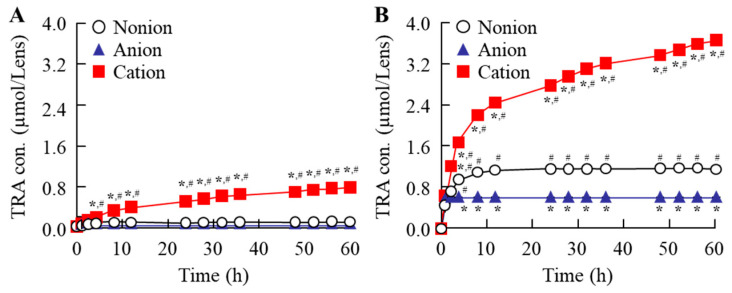
Changes in release profile of the TRA from the TRA-loaded CLs that used the stir- (**A**) and AC-methods (**B**). n = 3. * *p* < 0.05 vs. non-ion for each category. ^#^ *p* < 0.05 vs. anion for each category. The TRA in the cation-type CLs prepared when using the AC-method exhibited a sustained-release from all three types of the CLs. Moreover, the sustained-release of the TRA from the cation-type CLs that were prepared using the AC-method was significantly higher than that found for the other CLs.

**Figure 5:**
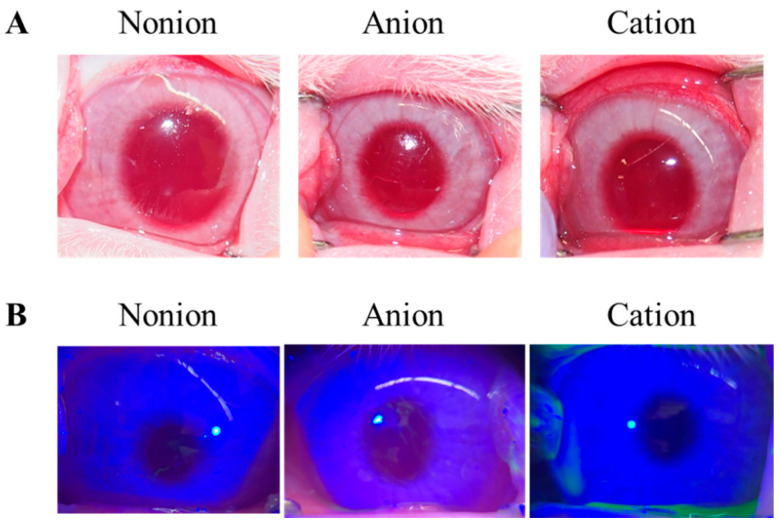
Corneal toxicity in the rabbits given the TRA-loaded CLs that were prepared using the AC-method. (**A**) Images of the eyes of the rabbits that were given the three types of the TRA-loaded CLs. The CLs were applied for 2 h. (**B**) Evaluation of the corneal wounds in the rabbits given the three types of the TRA-loaded CLs were performed using fluorescein. The CLs were repetitively applied to the rabbits for 8 h a day for 7 days, with the corneal wounds then subsequently monitored. There were three types of CLs used (non-ion-, anion-, and cation-type). There were no corneal wounds observed for any of the three types of the TRA-loaded CLs.

**Figure 6:**
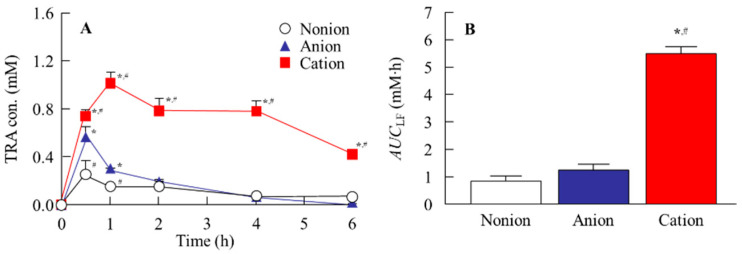
Profile (**A**) and *AUC*_LF_ (**B**) of TRA in the lacrimal fluid after the application of the three types of the TRA-loaded CLs prepared using the AC-method. There were three types of CLs used (non-ion-, anion-, and cation-type), with the TRA loaded to CLs using the AC-method. n = 5. * *p* < 0.05 vs. non-ion for each category. ^#^ *p* < 0.05 vs. anion for each category. The TRA-loaded CLs exhibited a sustained-release of TRA to the lacrimal fluid, with the TRA concentration in the lacrimal fluid the highest in the cation-type of the TRA-loaded CLs.

Figure 4 shows the release profile for TRA-loaded CLs. Although, the TRA release for the non-ion- and anion-type CLs plateaued at approximately 12 h and 4 h after the start of the experiment, respectively, the release of TRA from cation-type CL was observed for 60 h (Figure 4). Similar to that seen for the drug content in the CLs, the release of TRA from the cation-type CL prepared using the AC-method was the highest as compared to the other CLs that were prepared using the stir- and AC-methods. At 60 h, the TRA levels in the cation-type CLs that were prepared with the AC-method were 3.3- and 6.7-fold higher than the corresponding non-ion- and anion-type CL, respectively (Figure 4). In contrast, the drug release from the pre-lens and post-lens was similar regardless of the method that was used for the drug loading and the type of CL material (Table 1). Next, the CLs that were prepared using the AC-method were applied to the rabbits, with the CLs then investigated to determine if they caused corneal toxicity. Corneal wounds were not detected in any of the rabbit corneas that were administered any of the three types of TRA-loaded CLs (Figure 5). Figure 6 shows the changes in TRA concentration in the lacrimal fluid of the rabbits that were exposed to the TRA-loaded CLs that used the AC-method. The TRA in cation-type CLs exhibited a sustained release over 6 h, which was 6.7- and 4.6-fold higher than that of the corresponding non-ion- and anion-type CLs, respectively. In addition, the TRA concentration in the lacrimal fluid of the cation-type CL-applied rabbits demonstrated significantly greater sustainability compared to the instillation of unloaded TRA suspensions, since the TRA concentration in the lacrimal fluid of rabbit one hour after instillation of the TRA suspensions was 0.02 ± 0.006 mM (n = 5).

## 4. Discussion

Allergic conjunctivitis is an important cause of redness, severe swelling of the eyelids, conjunctivae, and a sensation of grittiness and burning. TRA is the primary drug used for the treatment of allergic conjunctivitis, with a sustainable TRA supply on the ocular surface expected to lead to the efficacious management of these disorders. The purpose of the present study was to evaluate the creation of sustainable TRA-releasing CLs when using three types of CLs that had a different water content and when using a soaking method under high-pressure and high-temperature. Moreover, our study results showed that the TRA-loaded cation-type CL sustainably released TRA to the lacrimal fluid in the rabbit.

In order to evaluate this release, we first attempted to load the TRA into CLs. Although the soaking method is widely used to load ocular drugs into CLs [34], this method can lead to a high burst release and low drug uptake [21]. Therefore, we investigated whether using a soaking method under high-pressure and high-temperature (AC-method) would enhance the loading of the TRA in the CLs in order to resolve the issues that limit this release when using the conventional soaking method (stir-method). All of the three types of CLs prepared using the stir- and AC-methods were transparent (Figure 2). Moreover, in all three types of these CLs, the TRA content in the CLs that were prepared using the AC-method were higher as compared to that found for the stir-method (Figure 3). In this current study, it was assumed that the dissolved TRA was loaded into the CLs, as these CLs were transparent (Figure 2). Moreover, it is known that the solubility of TRA is increased when under high-pressure and high-temperature conditions. Therefore, these findings suggested that the enhanced solubility and high-pressure conditions were related to the observed high TRA loading into the CLs. In addition, the TRA contents were strongly loaded into the cation-type lenses as compared to that observed for the non-ion-type and anion-type lenses (Figure 3). On the other hand, no particle aggregation was observed in the cation-type CLs during the present process. Furthermore, when the cation-type CL treated with AC method was evenly divided into eight segments, and the drug content in each segment were measured, no significant variation was observed (0.53 ± 0.03 µmol/each segment, mean ± standard deviation, n = 24). These results suggest that the drug is uniformly distributed throughout the CL.

We also demonstrated the release profile of the TRA from the CLs (Figure 4), and the TRA release in the non-ion- and anion-type CLs, which plateaued at 12 h and 4 h, respectively. In contrast, the most sustained release of TRA was observed for the cation-type CL that had been prepared using the AC-method, with the TRA released from the cation-type CL for 60 h. In addition, there was a similar TRA release from the pre-lens and post-lens (Figure 4). Previous studies have reported that the drug-loading and release behavior is dependent on the composition of the CLs [29,30], with the water content and ion charge particularly related to the affinity with the CLs and drug [28,29,30]. Both low and high affinity can cause a very fast release or no release, respectively, with these affinities found to be unbeneficial with regard to a sustained-release DDS. Hydrophobic interactions represent the entropy-driven association of nonpolar molecules in aqueous environments to minimize water disruption, and the interactions may relate the drug-loading and release behavior. However, the water content of three types of the CLs (non-ion-, anion-, and cation-type) used in this study were 38%, 58%, and 46%, respectively, with the difference in the water content of the CLs not related to the drug release. In contrast, the three types of the CLs used in this study also had different ion charges, along with the fact that TRA is a weakly acidic drug with carboxylic acid. The carboxylic acids dissociate under neutral conditions, forming a negative charge (R-COO^−^). Therefore, it has been hypothesized that the COO^−^ (carboxylic acids with negative charge) and the cationic part of the CL are easily bound compared to that for the non-ion-type and anion-type lenses under neutral conditions. Thus, the difference in the ionic charge may affect the loaded TRA content and release in these CLs. From these findings, differences in water content did not correlate with the drug loading, suggesting that the observed binding is likely governed primarily by electrostatic interactions. However, it remains important to further investigate whether these interactions are indeed predominantly electrostatic or whether other factors, such as hydrophobic interactions related to the CL materials, also play a role. These deeper discussions of drug–lens interaction mechanisms (e.g., possible hydrophobic interactions) help to further strengthen this study.

Subsequently, we examined the opacity and toxicity of the eyes of the rabbits that received the TRA-loaded CLs. Our findings indicated that there was no opacity or corneal damage observed in any of the three types of TRA-loaded CLs after repetitive application for one week (Figure 5). These results suggest that the TRA-loaded CLs prepared and utilized in this study can be used without affecting the vision, as these do not interfere with the transparency of the CLs. In this study, based on the results of fluorescence staining (Figure 5), quantitative analysis was performed using Image J. However, no detectable damage was observed in any of the samples. On the other hand, conducting histological testing in the future will be important for a more comprehensive evaluation of ocular safety.

It is important to confirm whether TRA can be sustainably released from a TRA-loaded CL in an in vivo study. Therefore, we investigated the TRA profile in the lacrimal fluid after the application of a TRA-loaded CL, since the use of a loaded-TRA is commonly used as a therapeutic drug for allergic conjunctivitis (Figure 6). In the in vivo study, the TRA release from the cation-type of the TRA-loaded CL when using the AC-method was prolonged compared to the corresponding non-ion- and anion-type of CLs, with a sustained drug release observed for more than 6 h after the application of the cation-type of CL prepared when using the AC-method (Figure 6). Taken together, this suggests that it is possible that the TRA-loaded cation-type of CL prepared using the AC-method will be able to improve the issues that are present when using the conventional soaking method, which include a low drug uptake and high burst release.

Further studies will need to be undertaken in order to investigate the therapeutic effect of TRA-loaded CLs on allergic conjunctivitis. Moreover, it is important to clarify the details of the mechanism associated with the drug loading of the TRA to the CLs when using the AC-method. Therefore, further studies will need to investigate which type of CLs (non-ion-, anion-, and cation-type) are more likely to load drugs with weakly acidic or basic properties when using the AC-method. In contrast, it is known that drugs released from the CLs into the post-lens tear film (POLTF) are mainly delivered into the anterior segment for the management of ocular diseases through the corneal route. Drugs released from CLs into the pre-lens tear film (PLTF) are dominantly absorbed into the conjunctiva, with the PLTF the only site for conjunctiva uptake during drug delivery by CLs [37,38]. We used a methacrylate cell designed for trans-corneal penetration studies [36,39] for investigating whether the drug is released from a TRA-loaded cation-type CL when using the AC-method on the PLTF or POLTF side. The results indicated that TRA in CLs was released from both the pre-lens and post-lens (Figure 4). Therefore, the application of drug-loaded CLs using the AC-method may be a useful therapy for ophthalmic disease in the anterior ocular segment, such as anterior uveitis and glaucoma, since drug release from the pre-lens may contribute to drug delivery into the lacrimal fluid, while release from the post-lens may facilitate trans-corneal delivery into the intraocular tissues. However, further studies will need to be undertaken in order to clarify the details of the therapeutic effect for the anterior ocular segment in drug-loaded CLs when using the AC-method.

## 5. Conclusions

The results of the present study demonstrated that TRA was easily loaded into a cation-type CL using the high-pressure and high-temperature soaking method (AC-method). The resulting TRA-loaded CL remained optically transparent. Moreover, sustained TRA release was observed for over 60 h from the TRA-loaded CL in the in vitro study. Furthermore, the TRA-loaded cation-type CL sustainably released TRA to the lacrimal fluid in the rabbit. These results provide useful information and will be beneficial in helping to develop an effective DDS to the ocular surface when using CLs.

## Figures and Tables

**Table 1:** Drug release (*AUC*_0–6 h_) from pre-lens and post-lens of non-ion-, anion-, and cation-type CLs prepared using the AC-method.

*AUC*_0–6 h_	TRA Release (µmol∙h/Lens)		
	Non-Ion	Anion	Cation
Pre-lens	60.3 ± 4.1 ^#^	30.0 ± 2.8 *	179.3 ± 8.1 *^,#^
Post-lens	63.4 ± 4.9 ^#^	34.6 ± 3.7 *	179.1 ± 8.5 *^,#^

Three types of CLs were used (non-ion-, anion-, and cation-type) (n = 6). * *p* < 0.05 vs. non-ion for each category. ^#^ *p* < 0.05 vs. anion for each category.

## Data Availability

The raw data supporting the conclusions of this article will be made available by the authors on request.

## References

[1] Komatsu H., Kojima M., Tsutsumi N., Hamano S., Kusama H., Ujiie A., Ikeda S., Nakazawa M. (1998). Study of the mechanism of inhibitory action of tranilast on chemical mediator release. Jpn. J. Pharmacol..

[2] Itoh F., Komatsu Y., Taya F., Isaji M., Momose Y., Suzawa H., Miyata H., Shibazaki T. (1993). Effect of tranilast ophthalmic solution on allergic conjunctivitis in guinea pigs. Nihon Yakurigaku Zasshi.

[3] Kumar A., Malviya R., Sharma P. (2011). Recent trends in ocular drug delivery: A short review. Am. J. Appl. Sci..

[4] Gaudana R., Ananthula H.K., Parenky A., Mitra A.K. (2010). Ocular drug delivery. AAPS J..

[5] Yung Y.H., Toda I., Sakai C., Yoshida A., Tsubota K. (2012). Punctal plugs for treatment of post-LASIK dry eye. Jpn. J. Ophthalmol..

[6] Pimenta A.F.R., Serro A.P., Colaço R., Chauhan A. (2018). Drug delivery to the eye anterior chamber by intraocular lenses: An in vivo concentration estimation model. Eur. J. Pharm. Biopharm..

[7] Lobo A.-M., Sobrin L., Papaliodis G.N. (2010). Drug delivery options for the treatment of ocular inflammation. Semin. Ophthalmol..

[8] Ludwig A. (2005). The use of mucoadhesive polymers in ocular drug delivery. Adv. Drug Deliv. Rev..

[9] Kakisu K., Matsunaga T., Kobayakawa S., Sato T., Tochikubo T. (2013). Development and efficacy of a drug-releasing soft contact lens. Investig. Ophthalmol. Vis. Sci..

[10] Costa V.P., Braga M.E.M., Duarte C.M.M., Alvarez-Lorenzo C., Concheiro A., Gil M.H., De Sousa H.C. (2010). Anti-glaucoma drug-loaded contact lenses prepared using supercritical solvent impregnation. J. Supercrit. Fluids.

[11] Ciolino J.B., Stefanescu C.F., Ross A.E., Salvador-Culla B., Cortez P., Ford E.M., Wymbs K.A., Sprague S.L., Mascoop D.R., Rudina S.S. (2014). In vivo performance of a drug-eluting contact lens to treat glaucoma for a month. Biomaterials.

[12] Ribeiro A.M., Figueiras A., Veiga F. (2015). Improvements in topical ocular drug delivery systems: Hydrogels and contact lenses. J. Pharm. Pharm. Sci..

[13] Lee D., Cho S., Park H.S., Kwon I. (2016). Ocular drug delivery through pHEMA-hydrogel contact lenses co-loaded with lipophilic vitamins. Sci. Rep..

[14] Mahaling B., Katti D.S. (2016). Understanding the influence of surface properties of nanoparticles and penetration enhancers for improving bioavailability in eye tissues in vivo. Int. J. Pharm..

[15] Bhatnagar S., Saju A., Cheerla K.D., Gade S.K., Garg P., Venuganti V.V.K. (2018). Corneal delivery of besifloxacin using rapidly dissolving polymeric microneedles. Drug Deliv. Transl. Res..

[16] Roy G., Galigama R.D., Thorat V.S., Mallela L.S., Roy S., Garg P., Venuganti V.V.K. (2019). Amphotericin B containing microneedle ocular patch for effective treatment of fungal keratitis. Int. J. Pharm..

[17] Gilger B.C., Mandal A., Shah S., Mitra A.K. (2014). Episcleral, intrascleral, and suprachoroidal routes of ocular drug delivery—Recent research advances and patents. Recent Pat. Drug Deliv. Formul..

[18] Costa J.R., Silva N.C., Sarmento B., Pintado M. (2015). Potential chitosan-coated alginate nanoparticles for ocular delivery of daptomycin. Eur. J. Clin. Microbiol. Infect. Dis..

[19] Destruel P.-L., Zeng N., Maury M., Mignet N., Boudy V. (2017). In vitro and in vivo evaluation of in situ gelling systems for sustained topical ophthalmic delivery: State of the art and beyond. Drug Discov. Today.

[20] Eljarrat-Binstock E., Domb A.J. (2006). Iontophoresis: A non-invasive ocular drug delivery. J. Control. Release.

[21] Guzman-Aranguez A., Colligris B., Pintor J. (2013). Contact lenses: Promising devices for ocular drug delivery. J. Ocul. Pharmacol. Ther..

[22] Li C.C., Chauhan A. (2007). Ocular transport model for ophthalmic delivery of timolol through p-HEMA contact lenses. J. Drug Deliv. Sci. Technol..

[23] Kim J., Chauhan A. (2008). Dexamethasone transport and ocular delivery from poly(hydroxyethyl methacrylate) gels. Int. J. Pharm..

[24] Hehl E.M., Beck R., Luthard K., Guthoff R., Drewelow B. (1999). Improved penetration of aminoglycosides and fluorozuinolones into the aqueous humour of patients by means of Acuvue contact lenses. Eur. J. Clin. Pharmacol..

[25] Ruben M., Watkins R. (1975). Pilocarpine dispensation for the soft hydrophilic contact lens. Br. J. Ophthalmol..

[26] Ribeiro A., Veiga F., Santos D., Torres-Labandeira J.J., Concheiro A., Alvarez-Lorenzo C. (2012). Hydrophilic acrylic hydrogels with builtin or pendant cyclodextrins for delivery of anti-glaucoma drugs. Carbohydr. Polym..

[27] Jones L., Powell C.H. (2013). Uptake and release phenomena in contact lens care by silicone hydrogel lenses. Eye Contact Lens.

[28] Schultz C.L., Poling T.R., Mint J.O. (2009). A medical device/drug delivery system for treatment of glaucoma. Clin. Exp. Optom..

[29] Peng C.-C., Kim J., Chanhan A. (2010). Extended delivery of hydrophilic drugs from silicone-hydrogel contact lenses containing vitamin E diffusion barriers. Biomaterials.

[30] Phan C.-M., Subbaraman L.N., Jones L. (2013). In vitro uptake and release of natamycin from conventional and silicone hydrogel contact lens materials. Eye Contact Lens.

[31] Franco P., De Marco I. (2021). Contact Lenses as Ophthalmic Drug Delivery Systems: A Review. Polymers.

[32] Rykowska I., Nowak I., Nowak R. (2021). Soft Contact Lenses as Drug Delivery Systems: A Review. Molecules.

[33] Maulvi F.A., Soni T.G., Shah D.O. (2016). A review on therapeutic contact lenses for ocular drug delivery. Drug Deliv..

[34] Minami M., Otake H., Nakazawa Y., Okamoto N., Yamamoto N., Sasaki H., Nagai N. (2021). Balance of drug residence and diffusion in lacrimal fluid determine ocular bioavailability in in situ gels incorporating tranilast nanoparticles. Pharmaceutics.

[35] Nagai N., Minami M., Deguchi S., Otake H., Sasaki H., Yamamoto N. (2020). An in situ gelling system based on methylcellulose and tranilast solid nanoparticles enhances ocular residence time and drug absorption into the cornea and conjunctiva. Front. Bioeng. Biotechnol..

[36] Otake H., Goto R., Ogata F., Isaka T., Kawasaki N., Kobayakawa S., Matsunaga T., Nagai N. (2021). Fixed-combination eye drops based on fluorometholone nanoparticles and bromfenac/levofloxacin solution improve drug corneal penetration. Int. J. Nanomed..

[37] Xu J., Xue Y., Hu G., Lin T., Gou J., Yin T., He H., Zhang Y., Tang X. (2018). A comprehensive review on contact lens for ophthalmic drug delivery. J. Control. Release.

[38] King-Smith P.E., Fink B.A., Hill R.M., Koelling K.W., Tiffany J.M. (2004). The thickness of the tear film. Curr. Eye Res..

[39] Nagai N., Ogata F., Otake H., Nakazawa Y., Kawasaki N. (2019). Energy-dependent endocytosis is responsible for drug transcorneal penetration following the instillation of ophthalmic formulations containing indomethacin nanoparticles. Int. J. Nanomed..

